# A Robust Procedure for Comparing Multiple Means under Heteroscedasticity in Unbalanced Designs

**DOI:** 10.1371/journal.pone.0009788

**Published:** 2010-03-29

**Authors:** Esther Herberich, Johannes Sikorski, Torsten Hothorn

**Affiliations:** 1 Institut für Statistik, Ludwig-Maximilians-Universität, München, Germany; 2 Deutsche Sammlung von Mikroorganismen und Zellkulturen GmbH, Braunschweig, Germany; University of East Piedmont, Italy

## Abstract

Investigating differences between means of more than two groups or experimental conditions is a routine research question addressed in biology. In order to assess differences statistically, multiple comparison procedures are applied. The most prominent procedures of this type, the Dunnett and Tukey-Kramer test, control the probability of reporting at least one false positive result when the data are normally distributed and when the sample sizes and variances do not differ between groups. All three assumptions are non-realistic in biological research and any violation leads to an increased number of reported false positive results. Based on a general statistical framework for simultaneous inference and robust covariance estimators we propose a new statistical multiple comparison procedure for assessing multiple means. In contrast to the Dunnett or Tukey-Kramer tests, no assumptions regarding the distribution, sample sizes or variance homogeneity are necessary. The performance of the new procedure is assessed by means of its familywise error rate and power under different distributions. The practical merits are demonstrated by a reanalysis of fatty acid phenotypes of the bacterium *Bacillus simplex* from the “Evolution Canyons” I and II in Israel. The simulation results show that even under severely varying variances, the procedure controls the number of false positive findings very well. Thus, the here presented procedure works well under biologically realistic scenarios of unbalanced group sizes, non-normality and heteroscedasticity.

## Introduction

Many research projects in Life Sciences employ comparative studies [1]–[5]. For example, biodiversity exploration such as in population genetics measures the properties of individuals belonging to different groups. Often, multiple groups each containing several individuals are compared for traits which may differ only quantitatively but not qualitatively. The scientific hypothesis under test is then most often formulated in terms of mean differences among at least two of these groups. However, choosing an appropriate statistical inference procedure in order to assess mean differences between multiple groups often poses a non-trivial challenge. First, for many statistically less well trained users it is hard to verify to which extent statistical procedures for comparing means are based on theoretical assumptions such as normality or homoscedasticity, i.e. homogeneous or equal variances among all groups. This may lead to misapplication of tests, which is often not even detected by reviewers or editors. Second, for a specific experiment an appropriate statistical procedure might not even be available from the statistical literature. This is the case when the researcher can not assume the variances to be equal under all experimental conditions. All previously suggested parametric procedures for comparisons of means, such as the methods by Tukey [6] and Dunnett [7], require homogeneous variances among all groups. Applying these methods under heteroscedasticity, which refers to heterogeneous or unequal variances among all groups, can result in extreme size violations. As a consequence, false positive results will be reported with a probability far higher than 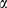, which is the a-priori chosen probability for wrongly rejecting a true null hypothesis. The situation is becoming even worse when unbalanced group sizes and/or non-normally distributed data are present. Unfortunately, unequal variances, non-normal data and unbalanced group sizes are realistic and hardly avoidable situations in biological research. A switch to non-parametric tests is not necessarily an option because even though they do not assume normality, they still assume that the shapes of the distributions are the same in all groups, which implies that variances are equal [8]. Several approaches for global comparison of several means under heteroscedasticity have been reported [9]–[12]. Yet no methods for multiple pairwise comparisons of means in presence of heteroscedasticity and potentially unequal sample sizes in the groups exist so far.

Hothorn *et al.* [13] introduced a statistical framework for simultaneous inference in general parametric models, which can be applied to a broad range of parametric models including ANOVA models. Neither homoscedasticity nor normality nor balanced group sizes are assumed, thus allowing for multiple comparisons in balanced and unbalanced models with arbitrary error distribution and hence arbitrary data distribution and variance structure. Pairwise comparisons of means can be tested simultaneously under control of the familywise error rate. The familywise error rate is the probability of falsely rejecting one or more hypothesis (i.e. finding a significant difference among the means of any two groups in the dataset even though there is actually no difference present) and is used as the standard measure for false positive results in multiple testing.

The aim of this paper is to advocate a new statistical method for the comparison of multiple means which does not suffer from increased false positive results the standard procedures will produce under non-normal heteroscedastic errors in unbalanced experimental designs. Asymptotic control of the familywise error rate for this procedure has been shown [13]. To assess the quality of the test under finite sample sizes, we examine the familywise error rate of the test under homoscedasticity as well as under heteroscedasticity for different error distributions in simulations and show that the familywise error rate is controlled. We also present the familywise error rate of procedures assuming homoscedasticity and show that the familywise error rate is not controlled under different forms of heteroscedasticity. In addition, we investigate the test's ability to find significant differences and therefore estimate the test's power, which is the probability of correctly rejecting a false hypothesis. We then reanalyze data from biodiversity research using this new procedure. In this research, the multiple cladogenic splits of evolutionary lineages (putative ecotypes) of the bacterium *Bacillus simplex* as an adaptational response to the microclimatically heterogeneous environment of “Evolution Canyon”, Israel, are being studied [14]–[17]. In this model population, unbalanced groups with frequently heterogeneous variances in their phenotypic properties are found. We apply the here presented method, which accounts for the existing heteroscedasticity. The analyzes are additionally conducted with methods requiring homogeneous variances. For several comparisons the results differ depending on whether heterogeneous variances are accounted for. When neglecting the heteroscedasticity, in several comparisons significant differences are found although they are actually not present or significant differences are not detected although they are present when the appropriate method is chosen. Results from simulations and the application to biodiversity research show how standard methods for multiple group comparison may fail under biologically realistic scenarios of heteroscedasticity and unbalanced groups, whereas the here presented method appears to be appropriate for such scenarios even in the situation of non-normal data. An implementation of the test procedure is provided in the **multcomp** package in the open-source-software R. We present R code which can be used to perform multiple comparisons of groups showing heterogeneous variances in the section “Computational Details”.

## Methods

### Model, Assumptions and Inference Procedures

We consider a one-way ANOVA model
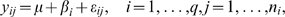
(1)where 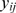 denotes the 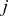
th observation in group 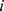, 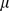 is the overall average, 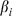 denotes the main effect in group 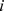 and 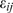 are random errors.

#### General linear hypotheses

To assess which particular groups differ concerning their means, we are interested in testing Tukey's all pairwise comparisons of group effects
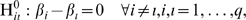
(2)or other post hoc comparisons simultaneously. To apply the inference procedure introduced by Hothorn *et al.* [13] these hypotheses have to be specified as general linear hypotheses of the model parameter vector 
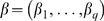
. The general linear hypothesis
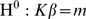
is set up by a matrix of linear functions 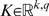, 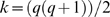 being the number of all pairwise comparisons. Each row of the matrix 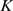 corresponds to one of the partial hypotheses 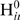. With the matrix 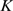 of the form
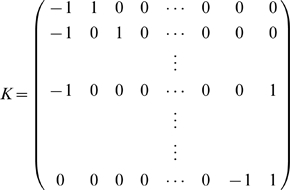
(3)and the right hand side of the hypotheses specified as 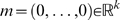, the general linear hypothesis corresponds to the partial hypotheses specified in equation (2). Further pairwise comparisons procedures like Dunnett's many-to-one comparisons can be specified by a corresponding matrix 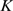.

#### Assumptions

We assume that an estimate 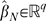 of the parameter vector 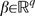 can be calculated based on 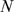 observations 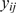 and that the estimate follows an asymptotic multivariate normal distribution 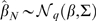. Additionally, a consistent estimation 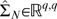 of the associated covariance matrix 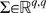 is required to be available. With these two assumptions fulfilled, the asymptotic distribution of the linear combinations 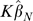 is available, which is a joint normal distribution 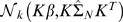 [13]. The deviation of the estimates 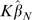 from the null hypothesis 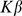 is standardized by 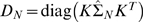. The 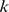 test statistics are defined in terms of these standardized deviations, i.e., 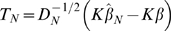 which again asymptotically follows a joint normal distribution: 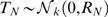 with 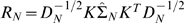. This distribution holds under heteroscedasticity or unequal sample sizes in the groups and is used as the reference distribution for the simultaneous inference on the comparisons specified in the general linear hypothesis.

#### Max-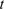 test

The max-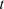 test provides the information which of the 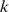 pairwise comparisons is significant [13]. It is based on 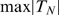, which is the maximum of the absolute values of the standardized test statistics 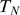. Under the null hypothesis the distribution function of this statistic is
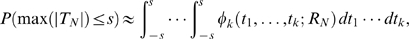
where 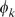 is the density function of the distribution 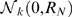. Adjusted single-step 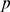-values, which control the familywise error rate, are
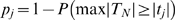
for the 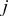
th partial hypothesis with 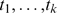 the components of the observed test statistic 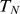. Approximate simultaneous 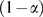-confidence intervals are given by
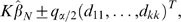
where 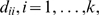 are the square roots the diagonal elements of 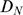.

#### Parameter estimation

In the derivation of the max-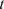 test we only assume that the parameter estimates are asymptotically multivariate normal with a consistent estimate of the associated covariance matrix being available. In an ANOVA model the group effects 
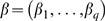
 are generally estimated by the ordinary least squares method. Under homoscedasticity the ordinary least squares parameter estimates are asymptotically normal and the ordinary least squares covariance estimation is a consistent estimation of the true covariance of the parameter estimates. Thus, both assumptions are fulfilled. In presence of unequal variances, the ordinary least squares parameter estimates are still asymptotically normal, while the covariance estimation obtained by the ordinary least squares estimation technique is inconsistent. Hence, a heteroscedastic consistent covariance estimation technique needs to be applied for simultaneous inference on the linear hypotheses. For small samples with a total number of observations up to 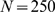 Long and Ervin suggest to use the covariance estimation HC3 introduced by MacKinnon and White [18], [19].

### Simulation

The inference procedure is based on the asymptotic distribution of the test statistic. To assess the quality of the max-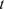 test in ANOVA models with finite sample sizes we investigated the familywise error rate and the power of the max-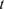 test in rather small samples by simulations. The familywise error rate must not exceed the a-priori defined level 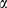, i.e., the probability of rejecting at least one true null hypothesis. If the familywise error rate is controlled, we are additionally interested in the power of the test, which measures the test's ability to find significant differences. For each false comparison the power is the probability of rejecting this false comparison.

We considered unbalanced one-way ANOVA models with 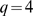 groups with equal variances 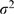 and normal data (A) and with heterogeneous variances with smaller variances in the smaller groups (B, D) and vice versa (C, E) both for normal and non-normal, right-skewed data. For the classical procedures, these special conditions of positive or negative pairing of group sizes and variances typically lead to conservative or liberal results, respectively.

A: 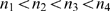 and 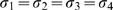, normal data.

B: 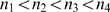 and 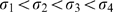, normal data.

C: 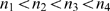 and 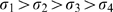, normal data.

D: 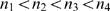 and 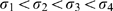, non-normal data.

E: 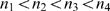 and 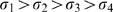, non-normal data.

For all pairwise comparisons of the group effects the familywise error rate and the power properties of the max-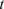 test using the covariance estimation HC3 were estimated and compared to the Tukey-Kramer test, which assumes equal variances among all groups.

#### Simulation parameters

Total sample sizes of 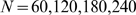 were considered with the 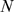 observations unbalancedly distributed to the four groups. The number of observations 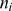 for each group 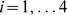 were defined as 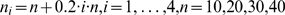, leading to 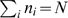. The overall mean was set to 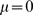 and all group effects were chosen equally 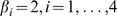. The random errors were independently normally distributed 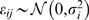 with group specific standard deviations 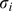. Standard deviations 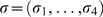 were chosen as 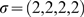 in model A, 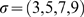 in model B, 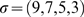 in model C, 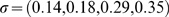 in model D and 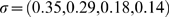 in model E.

#### Estimation of size and power

Datasets of size 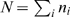 were simulated according to the considered models A to E. In each dataset all pairwise comparisons of the group effects were tested simultaneously by the max-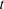 test accounting for heteroscedasticity and by the Tukey-Kramer test.

**Figure 1:**
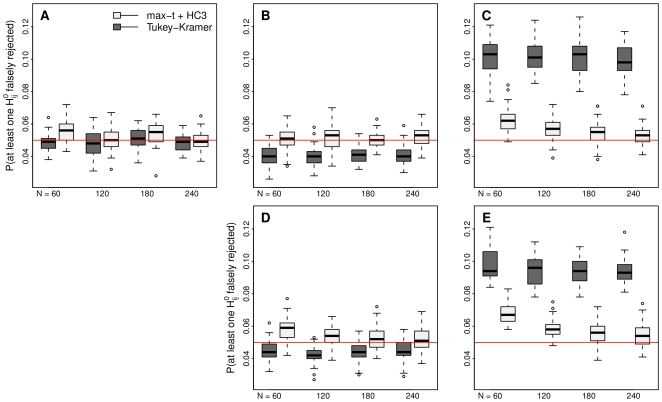
Familywise error rate of the simultaneous tests. Estimated familywise error rate of the max-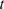 test using a heteroscedastic consistent covariance estimation (max-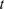
+HC3) and of the Tukey-Kramer test (Tukey-Kramer) assessing all pairwise comparisons of group effects in models under homoscedasticity (A), under heteroscedasticity with smaller variances in the smaller groups (B, D) and under heteroscedasticity with smaller variances in the larger groups (C, E) for normal data (A, B, C) and non-normal data (D, E). The total number of observations 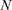 was unbalancedly distributed to the four groups. The horizontal red line indicates the a-priori defined level 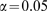.

To investigate the power of the tests the effects of groups 2 to 4 (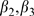 and 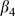) were kept equal while the effect of the first group 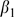 was chosen differently. Thus, the pairwise comparisons of 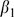 with each of the three other effects were false. For each of these false partial hypotheses the power of the max-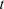 test and the Tukey-Kramer test were estimated by the proportion of correctly rejected partial hypotheses among 1000 datasets for increasing distances between 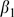 and 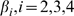. 41 values of distances 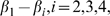 were considered. The familywise error rate was estimated by the proportion of datasets, in which at least one true partial hypothesis was falsely rejected. The same datasets were used for the analyzes of size and power leading to 41 estimated values of the familywise error rate each based on 1000 datasets. The distribution of the estimated familywise error rate is illustrated by the boxplots in Figure 1, where the boxplot for each setting is calculated from the 41 estimated values.

### Comparisons of fatty acid phenotypes of *Bacillus simplex* putative ecotypes under heteroscedasticity

The *B. simplex* population from “Evolution Canyons” I and II in Israel has recently developed to a model study of bacterial adaptation and speciation under heterogeneous environmental conditions [14]. These two canyons represent similar ecological sites, at a distance of 40 km, in which the orientation of the sun yields a strong sun-exposed and hot ‘African’ south-facing slope versus a rather cooler and mesic-lush ‘European’ north-facing slope within a distance of only 50–400 m. Phylogenetically, based on DNA sequences, the *B. simplex* population splits into two major groups GL1 and GL2. Interestingly, within each GL1 and GL2, further phylogenetic groups (or so called ‘putative ecotypes’) were observed which show a clear preference for either slope type [14], [15]. As a putative ecotype (PE) we regard a phylogenetic lineage whose members are adapted to specific ecological conditions [16], [20]. Whereas GL2 is composed of only PE1 and PE2, GL1 is made up of multiple PE (PE3–PE9) [15], [16]. In our quest to understand this characteristic slope type preference of the bacteria, we analyze physiological properties (phenotypes) that might be explanatory, such as temperature stress related phenotypes as a putative evolutionary adaptive response to the different temperatures on both slopes. For example, the physical integrity of the cell membrane at different temperatures is crucial for the cell survival. Here, the fatty acid composition of the cell membrane is of substantial importance. This was the motivation for a recent study on the contents of high- and low-temperature-tolerance-providing fatty acids (FAs) of the *B. simplex* ecotypes [17]. However, as the methods for the genetic characterization were improved in the meanwhile, leading to a re-shuffling of individuals into different groups (see also Table 3 of the supplemental material of [16]) and as the former fatty acid data were analyzed using the classical non-robust statistical tools [17] we take here the opportunity to reanalyze the experiment using the newly developed statistical tools presented in this manuscript. We focus specifically on the multiple ecotypes PE3 to PE9 from GL1 (we exclude PE8, as this ecotype is represented by only two bacterial strains).

Heteroscedasticity among the PE is assessed visually by boxplots, which illustrate the distribution of the FAs for the six PE. Analyzes are conducted both with methods assuming homoscedasticity and with methods accounting for heteroscedasticity to investigate in which way wrong conclusions are drawn when heterogeneous variances are ignored. We compute simultaneous confidence intervals for all pairwise differences of group effects to investigate which pairs of PE differ significantly concerning a specific growth condition of the bacteria [17]. These confidence intervals are calculated by the max-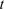 method using the ordinary least squares covariance estimation (assuming homoscedasticity), by the max-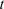 method using the heteroscedastic consistent covariance estimation HC3 as well as by the Tukey-Kramer method.

## Results

### Size and power of the max-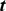 test

The estimated familywise error rates for all pairwise comparisons of group effects for both the max-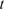 test using a heteroscedastic consistent covariance estimation and for the Tukey-Kramer test are illustrated in Figure 1. In the model with equal variances in all groups (model A) the estimated familywise error rate of the max-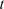 test is close to the a-priori chosen level of 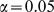 for either covariance estimation. With unequal variances and higher variances in the larger groups for both normal or non-normal data (models B and D), the Tukey-Kramer test is conservative while the estimated familywise error rate of the max-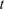 test using the heteroscedastic consistent covariance estimation is close to 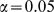 already for a total sample size of 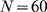. In the situation with higher variances in the smaller groups for both normal or non-normal data (models C and E), the usage of the Tukey-Kramer test results in serious violations of the familywise error rate. The familywise error rate of the max-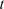 test using the consistent covariance estimation is liberal for a total sample size of 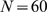 but close to 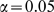 with increasing total sample size 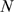.

**Figure 2:**
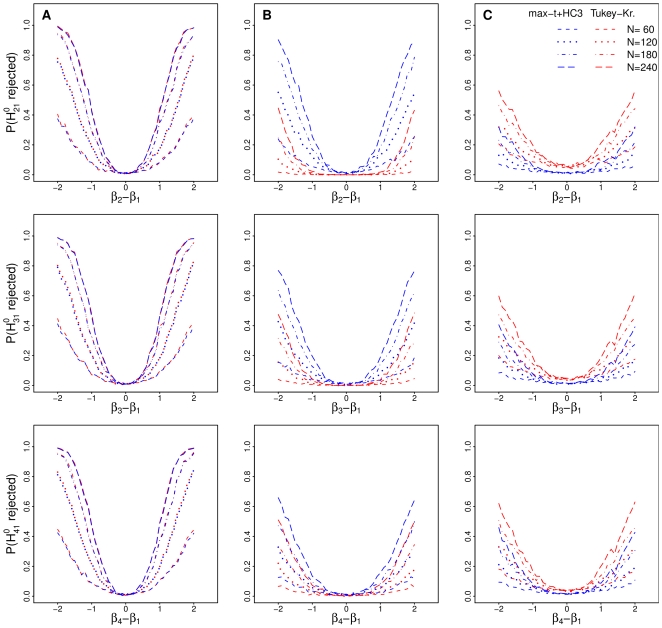
Power of the simultaneous tests. Comparison of the estimated power of the max-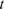 test using a heteroscedastic consistent covariance estimation (max-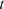
+HC3) and of the Tukey-Kramer test (Tukey) assessing all pairwise comparisons of group effects in models under homoscedasticity (A), under heteroscedasticity with smaller variances in the smaller groups (B) and under heteroscedasticity with smaller variances in the larger groups (C). The total number of observations 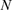 was unbalancedly distributed to the four groups.

Figure 2 shows the power curves of the max-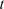 test for models A to C for the three pairwise comparisons of group effects 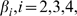 with 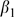, when the effects of the first group differs from the remaining effects. Under homoscedasticity (model A) the power of both multiple test procedures is almost identical for equivalent sample size 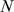. In model B, the power of the max-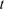 test is higher than the power of the Tukey-Kramer test. In model C the probability of discovering a false hypothesis is higher for the Tukey-Kramer test, but yet this test cannot be used because the familywise error rate is not controlled (Figure 1C).

### Comparisons of fatty acid phenotypes

**Figure 3:**
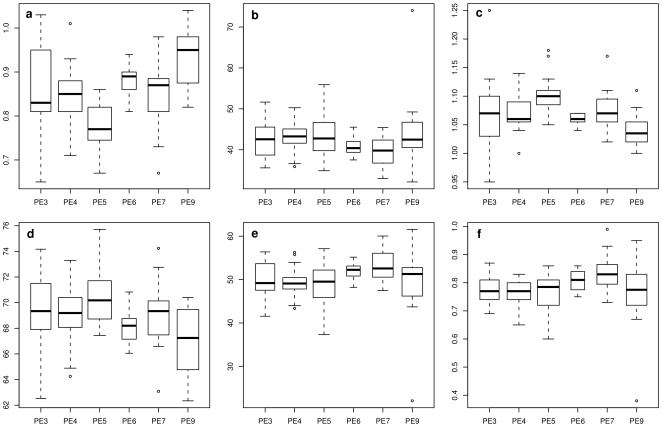
Distribution of the fatty acid content in six lineages (putative ecotypes, PE) of *B. simplex* for six different experimental conditions (a to f). Strains were grown on Trypticase Soy Broth Agar (Difco) for 24 hours at different temperatures. Harvesting of the cells, saponification, methylation, and extraction were performed according to instructions for fatty acid (FA) evaluation with the Sherlock Microbial Identification System (MIDI, Inc, Newark, USA). The samples were analyzed on an Agilent Technologies 6890N gas chromatograph. The FA content for each strain is reported as the percentage of FA among all FAs present. Fig. a and b sum up the high-temperature tolerance providing iso-branched FAs (i-14:0, i-15:0, i-16:0, i-17:0). Fig. a shows the ratio of these FA when the strains were grown at 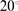
C versus 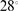
C. In Fig. b, the growth temperature was 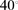
C. Fig. c to f sum up the cold-temperature tolerance providing anteiso-branched (ai-15:0, ai-17:0) and unsaturated FA (16:1 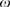
11c, 16:1 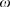
7c alcohol, i-17:1 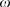
10c). The strains were grown at 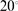
C (Fig. d) and 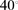
C (Figure e). Fig. c shows the ratio of 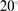
C/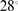
C, Fig. f the ratio of 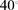
C/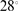
C. Further experimental details are described elsewhere [17].

**Figure 4:**
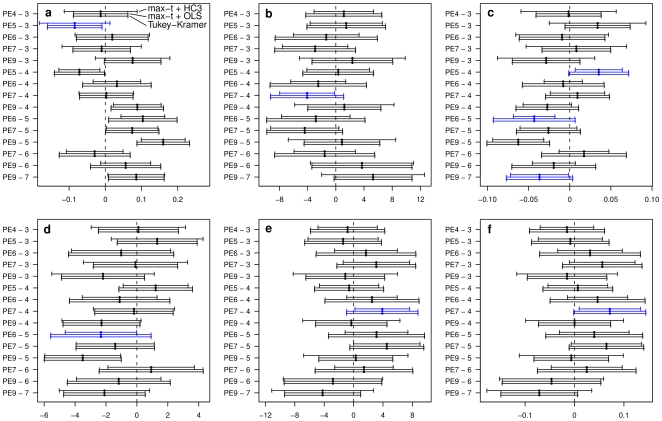
Simultaneous confidence intervals for all pairwise comparisons of group means. Intervals are computed by the max-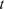 method accounting for heteroscedasticity using the heteroscedastic consistent covariance estimation HC3 (max-t+HC3), by the max-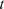 method assuming homoscedasticity using the ordinary least squares covariance estimation (max-t+OLS) and by the Tukey-Kramer method assuming homoscedasticity (Tukey-Kramer). The blue confidence intervals indicate the pairwise comparisons for which the decision of significant difference of the associated group means differs between the test procedures.

Figure 3 shows the distributions of high- and low-temperature-tolerance-providing FAs in six PE of *B. simplex* (PE3–PE9) for six different experimental conditions (Figures 3a to 3f). Variances differ considerably between the lineages within each type of experimental conditions. Thus, the validity of the results of the tests neglecting heteroscedasticity might be in question and attention should be drawn to the results of the max-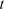 method accounting for heteroscedasticity. Results of the inference procedures assuming homoscedasticity (Tukey-Kramer method and max-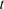 method using the ordinary least squares covariance estimation) are presented as well to show the extent of differences in the results (Figure 4).

The simultaneous confidence intervals for all pairwise differences of group effects for all six fatty acids calculated by the methods which assume homoscedasticity (Tukey-Kramer and ordinary max-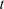 method) do not alter in any comparison of strains. In contrast, the width of the max-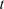 confidence intervals based on the heteroscedastic consistent covariance estimation is noticeably different, either narrower or wider.

Two PE are considered significantly different concerning their fatty acid content, if the associated simultaneous confidence interval does not include the zero. For several comparisons the decision of significant difference depends on the method chosen (simultaneous confidence intervals colored blue). When heterogeneous variances are neglected, a significant difference in the lineages PE3 and PE5 is found concerning the FAs (Figure 4a), which is not present when heteroscedasticity is accounted for. For the other FAs (Figure 4b to 4f) significantly differing lineages of *B. simplex* are not detected, when heteroscedasticity is ignored.

## Discussion

We described the application of the simultaneous inference procedure proposed by Hothorn *et al.* [13] to pairwise comparisons of means. By using an appropriate covariance estimation technique, the method can be used for multiple comparisons in presence of either equal or unequal group variances in balanced or unbalanced designs with arbitrary error distribution.

Simulations showed, that the familywise error rate is bound by the a-priori chosen level of 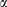 already for relatively small sample sizes in unbalanced designs with both normal or skewed error distributions and different kinds of pairing of group sizes and variance, whereas the Tukey-Kramer test can lead to false positive rates considerably higher than 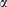. Even in situations where the Tukey-Kramer test does not lead to inflated false positive rates, the max-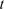 test is superior to the Tukey-Kramer test, as it has the higher power to detect existing differences in means.

Thus, the max-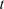 test for multiple comparisons of means using the heteroscedastic consistent covariance estimation in presence of unequal variances helps to avoid an increased number of false positive results. The procedure is implemented in the R [21] add-on package **multcomp** [22] utilizing an implementation of the HC3 estimator in package **sandwich** [23]. A short introduction along with an example is given in the Appendix.

### Computational Details

Install the R software from http://CRAN.R-project.org/. Then use the R software to install the packages multcomp and sandwich. The **multcomp** package in R provides a general implementation of the framework for global and simultaneous inference in parametric models. In this section we present R code which can be used to perform multiple comparisons of groups showing heterogeneous variances. Data has to be in a form with two columns, where the first column contains the grouping variable and the second column contains the quantitative values of the observations. This can be in a .txt, .csv or .Rda file, which can be imported in R by the functions read.table(), read.csv() and load() respectively, or by the R Commander. The example data used in the following correspond to the data underlying Figure 3A and 4A and are available in the **multcomp** package.

The example data fattyacid can be loaded by

> library(“multcomp”)

> data(“fattyacid”)

It contains the grouping variable (here the putative ecotype PE) in the first column and the fatty acid content (FA) by which the groups are to be compared in the second column:

> fattyacid

PE FA

1 PE9 0.95

2 PE9 0.95

3 PE9 1.04

4 PE9 1.01

5 PE9 0.86

.

.

.

91 PE3 0.83

92 PE3 1.02

93 PE3 0.89

The following R code performs all-pairwise comparisons of means of the fattyacid data. It can be applied to any other data by replacing fattyacid in the third line by the name of the object containing the data in the two-column way described above, and by replacing the variable names PE and FA by the names of the variables used in the dataset wherever PE and FA appear in the code.

> library(“sandwich”)

> amod <- aov(FA∼PE, data = fattyacid)

> amod_glht <- glht(amod, mcp(PE = “Tukey”), vcov = vcovHC)

> summary(amod_glht)

Simultaneous Tests for General Linear Hypotheses

Multiple Comparisons of Means: Tukey Contrasts

Fit: aov(formula = FA∼PE, data = fattyacid)

Linear Hypotheses:

Estimate Std. Error t value Pr(>|t|)

PE4 - PE3 =  = 0 −0.012820 0.034997 −0.366 0.99905

PE5 - PE3 =  = 0 −0.084398 0.033846 −2.494 0.13104

PE6 - PE3 =  = 0 0.019286 0.035760 0.539 0.99400

PE7 - PE3 =  = 0 −0.010048 0.038006 −0.264 0.99981

PE9 - PE3 =  = 0 0.075536 0.035783 2.111 0.28057

PE5 - PE4 =  = 0 −0.071579 0.019764 −3.622 0.00600 **

PE6 - PE4 =  = 0 0.032105 0.022887 1.403 0.71500

PE7 - PE4 =  = 0 0.002772 0.026258 0.106 1.00000

PE9 - PE4 =  = 0 0.088355 0.022923 3.854 0.00282 **

PE6 - PE5 =  = 0 0.103684 0.021085 4.917 <0.001 ***

PE7 - PE5 =  = 0 0.074351 0.024703 3.010 0.03678 *

PE9 - PE5 =  = 0 0.159934 0.021124 7.571 <0.001 ***

PE7 - PE6 =  = 0 −0.029333 0.027266 −1.076 0.88423

PE9 - PE6 =  = 0 0.056250 0.024072 2.337 0.18270

PE9 - PE7 =  = 0 0.085583 0.027297 3.135 0.02592 *

— Signif. codes: 0 ‘***’ 0.001 ‘**’ 0.01 ‘*’ 0.05 ‘.’ 0.1 ‘ ’ 1

(Adjusted p values reported – single-step method)

First, a common ANOVA model is fitted by the function aov(). The fitted model amod is then given to the function glht() which sets up the hypotheses to be tested (i.e. the multiple contrasts of means). The argument vcov = vcovHC specifies the use of the heteroscedastic consistent covariance estimation HC3 accounting for the heterogeneous variances. The function vcovHC() and further heteroscedastic consistent sandwich covariance estimation functions are provided in the package **sandwich**. For multiple comparisons by the max-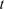 method in the situation of homogeneous variances the argument vcov = vcovHC of the function glht() has to be omitted.

Adjusted 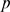-values assuring that the familywise error rate is not larger than 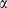 are computed by the summary() function. For each pairwise comparison the adjusted 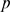-values are given in the last column of the output (column headed ‘Pr(>|t|)’). An adjusted 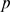-value of smaller than the a-priori chosen value of 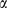 indicates a significant difference of the corresponding group means. We here find six significant differences on the level 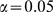. Significance is marked by asterisks at the end of the associated row.

Simultaneous confidence intervals for each difference of means can be computed by

> confint(amod_glht)

Simultaneous Confidence Intervals

Multiple Comparisons of Means: Tukey Contrasts

Fit: aov(formula = FA∼PE, data = fattyacid)

Estimated Quantile = 2.8935

95% family-wise confidence level

Linear Hypotheses:

Estimate lwr upr

PE4 - PE3 =  = 0 −0.012820 −0.114083 0.088444

PE5 - PE3 =  = 0 −0.084398 −0.182332 0.013535

PE6 - PE3 =  = 0 0.019286 −0.084185 0.122756

PE7 - PE3 =  = 0 −0.010048 −0.120016 0.099921

PE9 - PE3 =  = 0 0.075536 −0.028002 0.179074

PE5 - PE4 =  = 0 −0.071579 −0.128765 −0.014393

PE6 - PE4 =  = 0 0.032105 −0.034117 0.098328

PE7 - PE4 =  = 0 0.002772 −0.073204 0.078748

PE9 - PE4 =  = 0 0.088355 0.022027 0.154683

PE6 - PE5 =  = 0 0.103684 0.042676 0.164693

PE7 - PE5 =  = 0 0.074351 0.002874 0.145828

PE9 - PE5 =  = 0 0.159934 0.098812 0.221057

PE7 - PE6 =  = 0 −0.029333 −0.108227 0.049560

PE9 - PE6 =  = 0 0.056250 −0.013400 0.125900

PE9 - PE7 =  = 0 0.085583 0.006601 0.164565

where the entries of the columns headed ‘lwr’ (lower) and ‘upr’ (upper) give a lower and an upper bound for the confidence interval of each contrast.

> plot(confint(amod_glht))

visualizes the simultaneous confidence intervals.

The given R Code performs Tukey's all pairwise comparisons of means. Dunnett's many-to-one contrasts comparing several groups each with a reference group can be tested by replacing the argument mcp(PE = “Tukey”) by mcp(PE = “Dunnett”) in the function glht(). Arbitrary other multiple contrasts of group means can be described symbolically, e.g. by replacing the argument mcp(PE = “Tukey”) by mcp(PE = c(“PE4 - PE3 = 0”,

“PE5 - PE3 = 0”,

“PE9 - PE5 = 0”)),

for comparisons of means of groups 4 and 3, 5 and 3, and 9 and 5.

Further details to the above listed R code are available at http://CRAN.R-project.org/package=multcomp.

The simulation results can be reproduced using the R transcript file available via

> file.show(system.file(“multcomp_VA.R”, package = “multcomp”))
